# Acute refractory hypoxemia after chest trauma reversed by high-frequency oscillatory ventilation: a case report

**DOI:** 10.1186/1752-1947-7-186

**Published:** 2013-07-15

**Authors:** Emmanuel Charbonney, Jennifer LY Tsang, Jeffrey Wassermann, Neill KJ Adhikari

**Affiliations:** 1 Centre de Santé et de Services Sociaux de Trois-Rivières, 1991 Boul. du Carmel, Trois-Rivières, QC G8Z 3R9, Canada; 2 Sunnybrook Health Sciences Centre, 2075 Bayview Avenue, Toronto, ON M4N 3M5, Canada; 3 St Michael’s Hospital, 30 Bond Street, Toronto, ON M5B 1W8, Canada

## Abstract

**Introduction:**

Polytrauma often results in significant hypoxemia secondary to direct lung contusion or indirectly through atelectasis, systemic inflammatory response, large volume fluid resuscitation and blood product transfusion. In addition to causing hypoxemia, atelectasis and acute lung injury can lead to right ventricular failure through an acute increase in pulmonary vascular resistance. Mechanical ventilation is often applied, accompanied with recruitment maneuvers and positive end-expiratory pressure in order to recruit alveoli and reverse atelectasis, while preventing excessive alveolar damage. This strategy should lead to the reversal of the hypoxemic condition and the detrimental heart–lung interaction that may occur. However, as described in this case report, hemodynamic instability and intractable alveolar atelectasis sometimes do not respond to conventional ventilation strategies.

**Case presentation:**

We describe the case of a 21-year-old Caucasian man with severe chest trauma requiring surgical interventions, who developed refractory hypoxemia and overt right ventricular failure. After multiple failed attempts of recruitment using conventional ventilation, the patient was ventilated with high-frequency oscillatory ventilation. This mode of ventilation allowed the reversal of the hemodynamic effects of severe hypoxemia and of the acute cor pulmonale. We use this case report to describe the physiological advantages of high-frequency oscillatory ventilation in patients with chest trauma, and formulate the arguments to explain the positive effect observed in our patient.

**Conclusions:**

High-frequency oscillatory ventilation can be used in the context of a blunt chest trauma accompanied by severe hypoxemia due to atelectasis. The positive effect is due to its capacity to recruit the collapsed alveoli and, as a result, the relief of increased pulmonary vascular resistance and subsequently the reversal of acute cor pulmonale. This approach may represent an alternative in case of failure of the conventional ventilation strategy.

## Introduction

Polytrauma and chest trauma often result in significant hypoxemia secondary to direct or indirect lung injury. Indirect lung injury can result from the systemic inflammatory response to the trauma itself or to the interventions such as large volume fluid resuscitation and blood product transfusion [1]. However, direct injury to the parenchyma often leads to bleeding, contusion and impaction of secretions with subsequent airway obstruction. The consequence is the development of multiple areas of atelectasis. Besides primary intervention, such as pneumothorax or hemothorax drainage, the management of chest injury and hypoxemia requires oxygen supplementation and often mechanical ventilation.

Ventilatory strategies for trauma patients are similar to those for patients with acute respiratory distress syndrome (ARDS). It is generally accepted to apply mechanical ventilation according to the open lung concept, consisting of intermittent recruitment maneuvers and positive end-expiratory pressure (PEEP), along with a low tidal volume to limit end-inspiratory transalveolar pressures [2]. The goals of this strategy are to achieve better oxygenation through alveoli recruitment, while preventing excessive alveolar damage. In addition to causing a mismatch in the ventilation to perfusion ratio, atelectasis and lung injury can lead to right ventricular failure through an acute increase in afterload secondary to increased pulmonary vascular resistance [3].

The use of high-frequency oscillatory ventilation (HFOV) has been reported in trauma patients as a safe rescue therapy [4], including in patients sustaining pulmonary contusions [5,6]. We describe a case of an injured patient with severe blunt chest trauma requiring surgery, who developed refractory hypoxemia during surgery. After multiple failed attempts of recruitment using conventional ventilation (CV) in the Intensive Care Unit (ICU), the patient’s severe hypoxemia was reversed by HFOV. As far as we are aware, this is the first case report of HFOV used as an extremely early rescue therapy shortly after admission to the ICU from the Operating Room (OR). We use this case report to describe the physiological advantages of HFOV in patients with chest trauma and formulate the arguments to explain the positive effect observed in our patient.

## Case presentation

A 21-year-old unrestrained Caucasian man, involved in a motor vehicle collision, was ejected after multiple rollovers and was found lying on the ground. His initial vital signs in the field were stable and he was awake and conversing. He was brought to the Emergency Room (ER) by ambulance at 2:30 a.m. His injuries included an open femur fracture, lower limb lacerations and blunt chest trauma.

While he was being assessed, he required 2L of crystalloids and two units of packed red blood cell (PRBC) transfusion to maintain his blood pressure. The focused assessment with sonography for trauma was negative and the first chest radiograph was unremarkable. After he was intubated in the ER for respiratory distress, a whole body computed tomography scan was performed. This examination revealed multiple vertebral fractures (T5 to T8) and a large hemothorax on the right side for which a chest tube was placed. After draining 800mL of blood, the drainage stabilized. The patient’s arterial blood pressure was maintained at 120/75mmHg without vasopressor support, but he was anuric. He was then brought to the ICU at 3:45 a.m. for close observation and continuous resuscitation. One hour later, he became hypotensive (systolic blood pressure <90mmHg) and tachycardic (heart rate >110 beats per minute), which coincided with the rapid drainage of 1.5L of frank blood from his chest tube. He was transferred to the OR for emergent thoracotomy.

The source of the bleeding was the posterior mediastinum in the area of the vertebral fractures. The bleeding was still active and had breached the pleural cavity. To allow for operative access, the anesthetist placed a right bronchial blocker to deflate the right lung. Paravertebral and intercostal vessels were then controlled. The severe bleeding and ongoing coagulopathy necessitated the transfusion of 24 units of PRBCs, 12 units of frozen plasma and 19 units of cryoprecipitate. He was also given recombinant Factor VIIa (7.2mg). Mixed respiratory and metabolic acidosis ensued. Inotropes and vasopressors (epinephrine and norepinephrine), intravenous bicarbonate and calcium gluconate were administered due to hemodynamic instability.

Because of intolerance to single-lung ventilation, the bronchial blocker was removed on two occasions. After the second pullback of the bronchial blocker, the patient became difficult to ventilate, with high airway pressures and low tidal volume. The patient was hypoxemic with a partial pressure of oxygen in arterial blood (PaO_2_) of 54mmHg while on 100% oxygen (Table 1). His central venous pressure (CVP) rose above 30mmHg. He was paralyzed, open lung ventilation was applied (PEEP = 15cm of water (H_2_O), ratio of inspiratory to expiratory time = 1), and a chest tube was placed on the left side. Due to increasing airway pressures and the high volume of fluid resuscitation administered, poor extrapulmonary compliance due to abdominal compartment syndrome was suspected. A decompressive laparotomy was performed, which led to partial improvement.

**Table 1:** Respiratory, hemodynamic and laboratory parameters

	**Day 0**	**Day 0**	**Day 0**	**Day 0**	**Day 0**	**Day 0**	**Day 0**	**Day 0**	**Day 0**	**Day1**
Time	02:37	03:50	05:16	06:00	9:00^**#**^	09:57	10:40	13:00	17:00	09:20
Location	ER	ER	ICU	OR	OR	ICU	ICU	ICU	ICU	ICU
FiO_2_	0.8	1.0	1.0	1.0	1.0	1.0	1.0	0.5	0.5	0.6
pH	7.03	NA	7.01	6.84	6.83	6.86	6.87	7.0	7.11	7.11
PaO_2_ (mmHg)	42	Sat. 89%	86	67	26	39	33	61	105	74
PaCO_2_ (mmHg)	62	NA	55	69	67	54	64	50	43	53
PEEP and/or mean airway pressure (cmH_2_O)*		5	12	18	15	15	*38	*39	*36	*35
Mean arterial pressure (mmHg)	55	85	45	70	65	55	60	65	66	75
CVP (mmHg)	NA	NA	16	15	40	30	NA	18	15	15
Lactate (mmol/L)	5.9	NA	7.5	8.6	7.3	8.4	9.8	10.2	6.4	4.2
Hemoglobin (g/L)	150	NA	56	67	67	81	NA	113	119	100
Norepinephrine (μg/kg /minute)	0	0	0.45	2	0.4	NA	0.8	0.7	0.9	1.5
Epinephrine (μg/kg/minute)	0	0	0	0	0.5	NA	0.4	–	–	–

^#^Arrival in the ICU; * high-frequency oscillatory ventilation started at 10:40; –, discontinued;

*CVP* central venous pressure, *ER* Emergency Room, *FiO*_*2*_ fraction of inspired oxygen, *H*_*2*_*O* water, *ICU* Intensive Care Unit, *NA* not available, *OR* Operating Room, *PaCO*_*2*_ partial pressure of carbon dioxide in arterial blood, *PaO*_*2*_ partial pressure of oxygen in arterial blood, *PEEP* positive end-expiratory pressure, *Sat* saturation.

**Figure 1:**
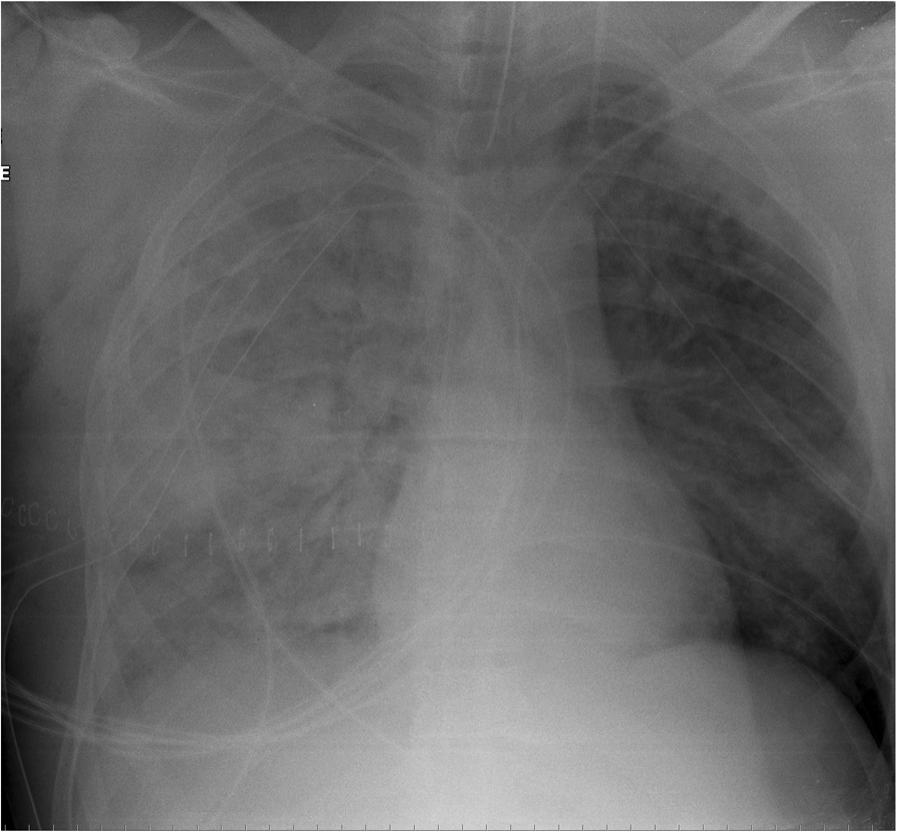
Chest X-ray taken at the admission in the intensive care unit, showing diffuse bilateral lung infiltrates and extensive airspace disease on the right lung.

When the patient was transferred back to the ICU after 3.5 hours in the OR, his hypoxemia had worsened (Table 1) with a persistent oxygen saturation of <50%. The chest radiograph showed bilateral lung infiltrates, with extensive airspace disease on the right lung (Figure 1). Multiple recruitment maneuvers were done. For each maneuver, the ventilator was set to pressure support mode with the pressure support level at 0cmH_2_O and the pressure alarm and apnea alarms were adjusted to 50cmH_2_O and 60 seconds respectively. The PEEP was then increased to 40cmH_2_O for 40 seconds while maintaining the inspired fraction of oxygen at 1.0. After each maneuver, PEEP was decreased to various levels down to 15cmH_2_O and ultimately left at 15cmH_2_O due to progressive desaturation and hemodynamic instability at higher levels. Given the inability to prone the patient due to extreme hemodynamic instability and the presence of multiple chest tubes, inhaled nitric oxide (up to 60 parts per million) was started, but without improvement in oxygenation. A bedside transthoracic echo showed right ventricular dilatation while the patient was on inotropes and vasopressors. In addition, the patient remained anuric despite prescription of diuretics.

The ICU team next decided to ventilate the patient with HFOV (Sensormedics 3100B, CareFusion, San Diego, USA). The initial settings were: fraction of inspired oxygen (FiO_2_) 100%, bias flow, 40L/minute; frequency, 4Hz (given the extreme acidosis); inspiration time, 33%; pressure amplitude of oscillation 83cmH_2_O, and mean airway pressure 38cmH_2_O (5cm H_2_O above mean airway pressure on CV). After 45 minutes, the PaO_2_ had reached 75mmHg and CVP dropped to 18mmHg. Two hours later, the FiO_2_ was titrated down to 50% and the epinephrine infusion was discontinued with maintenance of norepinephrine. Within a few hours, continuous veno-venous hemodialysis was started due to persistent anuria and hyperkalemia.

**Figure 2:**
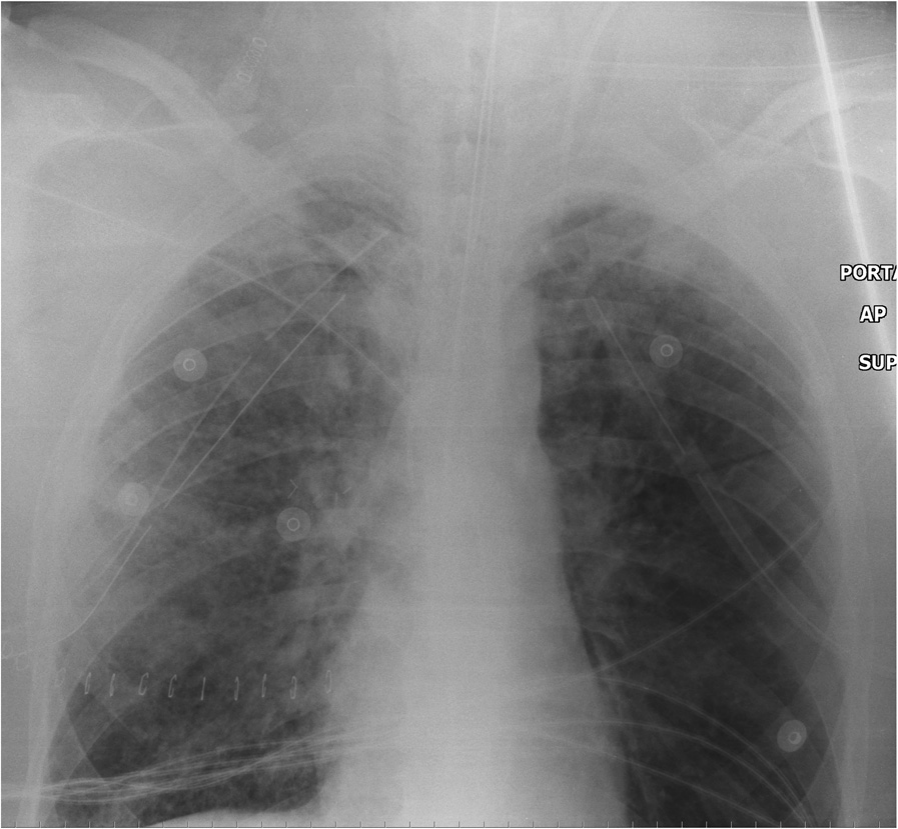
**Chest X-ray 48 hours after intensive care unit admission.** We see a clear improvement, with decreased bilateral infiltrates, compared with previous X-ray.

The next day, the patient still met criteria for ARDS, but his oxygenation had stabilized and radiographic improvement was visible at 48 hours (Figure 2). The patient was switched to CV 96 hours later. In the meantime, he developed rhabdomyolysis and elevated liver enzymes and required renal replacement therapy for 13 days; he also had two episodes of sepsis. He was weaned from the ventilator after 41 days, transferred to the ward after 51 days and discharged from the hospital 93 days after his injury.

## Discussion

HFOV has usually been described and used as “rescue therapy” in intractable ARDS with severe hypoxemia, and its use as early ventilatory support for ARDS is not supported by recent clinical trials [7,8]. Despite the description of “early” use (0 to 14 days after injury) of HFOV in trauma patients in retrospective series [4,5], no report of extreme early use has been described.

To the best of our knowledge, the case we describe above is the first report of HFOV as an “extreme early rescue therapy” for refractory hypoxemia after blunt chest trauma. The consequences of the chest trauma and surgery (mainly alveolar atelectasis, but also alveolar edema) had led to severe hypoxemia and adverse heart–lung interactions that were not reversible with CV. The efficacy of recruitment on the collapsed lung provides the explanation for the positive outcome observed once HFOV was started.

After blunt chest trauma, followed by the additional hit of intra-thoracic surgery, the ventilatory and radiographic abnormalities are mainly the consequences of alveolar collapse (atelectasis) and edema due to lung contusion and surfactant alteration [9]. A component of permeability edema due to trauma, polytransfusion, emergency thoracic surgery, and high-pressure ventilation in the OR is probable. However, we believe that the main pathophysiological process was acute cor pulmonale. The latter pathophysiological explanation is supported not only by the elevated CVP and the need of inotropes and vasopressors, but also by the rapid decrease of FiO_2_ requirement, CVP and inotropic requirement, after lung recruitment by the HFOV. However, measurement of changes in lung mechanics (compliance and resistance) was not possible while the patient was receiving HFOV.

**Figure 3:**
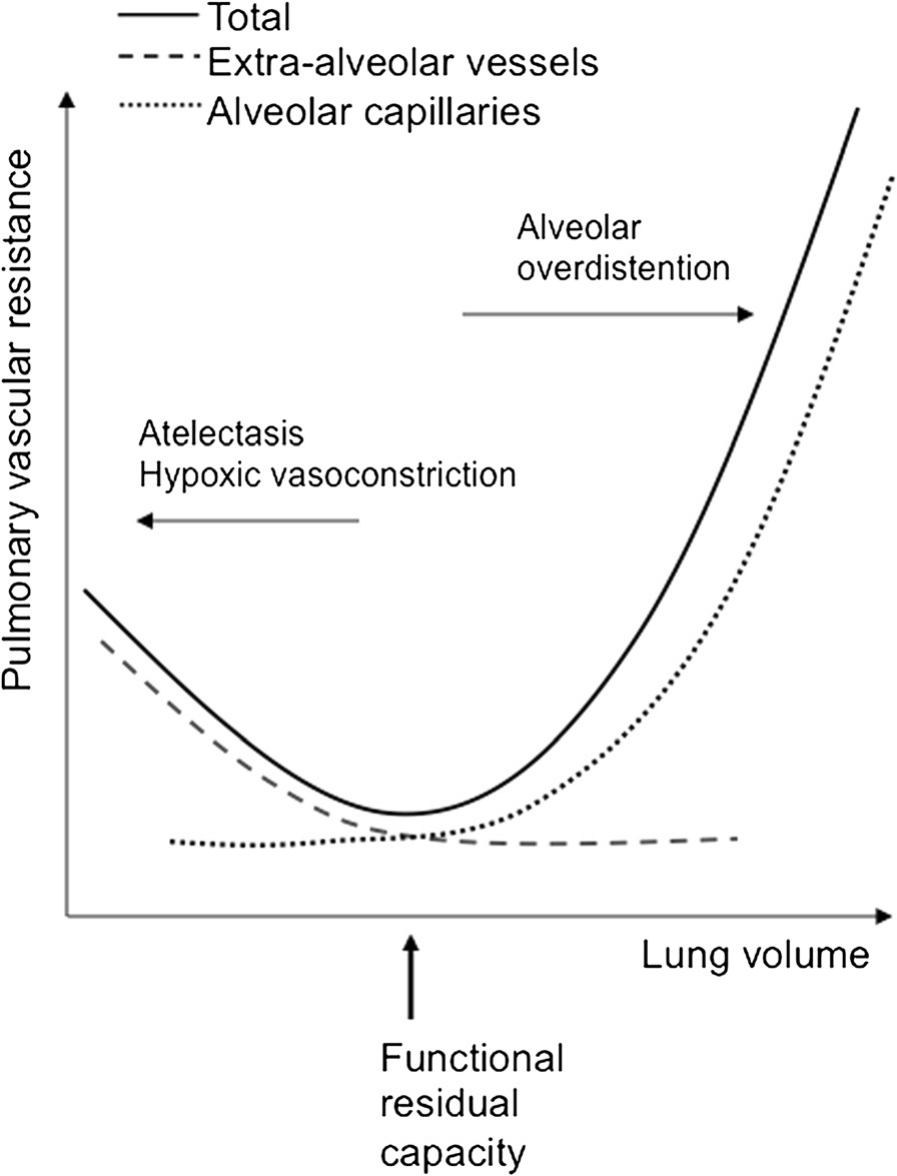
**Representation of the relationship between the lung volume and the lung vascular resistance.** Our patient had extensive lung atelectasis due to the trauma and surgery (arrow toward the left), and the attempt of recruitment of the collapsed alveoli with conventional ventilation lead to overinflation of the lung in other areas (arrow toward the right). Adapted from Simmons, HD *et al*., *Circulation Research*, 1961 [10]. Promotional and commercial use of the material in print, digital or mobile device format is prohibited without the permission from the publisher Lippincott Williams & Wilkins. Please contact http://journalpermissions@lww.com for further information.

The physiological rationale for the use and success of HFOV in this case rests on the relation between lung volume and vascular resistance, as represented by the “U-shaped” curve described by D.H. Simmons *et al*. in 1961 (Figure 3) [10]. Two factors contributed to increased vascular resistance: 1) the extensive alveolar atelectasis due to trauma and surgery (arrow toward the left), and 2) the overinflation of the “non-dependent” zones produced by the CV, due to unsuccessful recruitment of the other collapsed alveoli (arrow toward the right).

The theoretical advantage of HFOV is the presence of a high continuous distending pressure (mean airway pressure) that decreases from the ventilator to the distal trachea and is associated with less lung inflammation compared to CV with the same measured mean airway pressure [11]. In selected cases, HFOV may recruit lung more rapidly at comparable distending pressures due to prolonged elevation of pressure over time, associated with incremental pressure distribution and oscillations. These characteristics may better exploit pressure-volume hysteresis of collapsing unstable alveolar units than CV, through avoidance of the opening-closing phenomenon [12,13]. If HFOV achieves faster recruitment than CV, then the deleterious effects of HFOV on right ventricular function [14] may be reduced or avoided altogether.

Once the effect of afterload reduction by opening the lung and reducing the pulmonary arterial resistance is achieved, acute cor pulmonale should resolve [15]. However, we acknowledge that further improvement of our patient’s oxygenation was due to correction of the hypervolemic state with the continuous veno-venous hemodialysis.

## Conclusions

In conclusion, this patient presented with extreme hypoxemia in the context of blunt chest trauma accompanied by atelectasis and ARDS, with subsequent acute cor pulmonale. CV was ineffective in reversing the process. The introduction of HFOV allowed the recruitment of collapsed alveoli and, as a result, the rapid relief of increased pulmonary vascular resistance and subsequently the reversal of acute cor pulmonale. This illustrates the potentially beneficial role of rapid HFOV-mediated lung recruitment in desperate situations on both oxygenation and right ventricular function, notwithstanding recent evidence from landmark clinical trials finding no benefit [8] or harm [7] from the routine application of HFOV in patients with early ARDS.

## Consent

Written informed consent was obtained from the patient for publication of this case report and accompanying images. A copy of the written consent is available for review by the Editor-in-Chief of this journal.

## Competing interests

The authors declare that they have no competing interests.

## Authors' contributions

EC and JW contributed to the acquisition, the analysis, and the interpretation of the data. They drafted the article. JLT and NKJA contributed to the interpretation of the data, drafting of the article, and revision for intellectual content. All authors read and approved the final version of the manuscript.

## References

[B1] Calfee CS Eisner MD Ware LB Thompson BT Parsons PE Wheeler AP Korpak A Matthay MA Trauma-associated lung injury differs clinically and biologically from acute lung injury due to other clinical disorders Crit Care Med 2007 35 2243 2250 17944012 10.1097/01.ccm.0000280434.33451.87 PMC2765812

[B2] Schreiter D Reske A Stichert B Seiwerts M Bohm SH Kloeppel R Josten C Alveolar recruitment in combination with sufficient positive end-expiratory pressure increases oxygenation and lung aeration in patients with severe chest trauma Crit Care Med 2004 32 968 975 10.1097/01.CCM.0000120050.85798.38 15071387

[B3] Vieillard-Baron A Schmitt JM Augarde R Fellahi JL Prin S Page B Beauchet A Jardin F Acute cor pulmonale in acute respiratory distress syndrome submitted to protective ventilation: incidence, clinical implications, and prognosis Crit Care Med 2001 29 1551 1555 10.1097/00003246-200108000-00009 11505125

[B4] Briggs S Goettler CE Schenarts PJ Newell MA Sagraves SG Bard MR Toschlog EA Rotondo MF High-frequency oscillatory ventilation as a rescue therapy for adult trauma patients Am J Crit Care 2009 18 144 148 10.4037/ajcc2009303 19255104

[B5] Funk DJ Lujan E Moretti EW Davies J Young CC Patel MB Vaslef SN A brief report: the use of high-frequency oscillatory ventilation for severe pulmonary contusion J Trauma 2008 65 390 395 10.1097/TA.0b013e31817f283f 18695477

[B6] Gradišek MJ Gradišek P Kremžar B High frequency oscillatory ventilation as the most appropriate treatment for life threatening thoracic trauma Signa Vitae 2012 7 46 48

[B7] Ferguson ND Cook DJ Guyatt GH Mehta S Hand L Austin P Zhou Q Matte A Walter SD Lamontagne F Granton JT Arabi YM Arroliga AC Stewart TE Slutsky A Meade MO High-frequency oscillation in early acute respiratory distress syndrome N Engl J Med 2013 368 9 795 805 10.1056/NEJMoa1215554 23339639

[B8] Young D Lamb SE Shah S Mackenzie I Tunnicliffe W Lall R Rowan K Cuthbertson BH High-frequency oscillation for acute respiratory distress syndrome N Engl J Med 2013 368 9 806 813 10.1056/NEJMoa1215716 23339638

[B9] Groeneveld AB Increased permeability-oedema and atelectasis in pulmonary dysfunction after trauma and surgery: a prospective cohort study BMC Anesthesiol 2007 7 7 10.1186/1471-2253-7-7 17620115 PMC1939984

[B10] Simmons DH Linde LM Miller JM O'Reilly RJ Relation between lung volume and pulmonary vascular resistance Circ Res 1961 9 2 465 471 10.1161/01.RES.9.2.465

[B11] Muellenbach RM Kredel M Said HM Klosterhalfen B Zollhoefer B Wunder C Redel A Schmidt M Roewer N Brederlau J High-frequency oscillatory ventilation reduces lung inflammation: a large-animal 24-h model of respiratory distress Intensive Care Med 2007 33 1423 1433 10.1007/s00134-007-0708-x 17563879

[B12] Ferguson N Slutsky A Last word on point: counterpoint: high-frequency ventilation is/is not the optimal physiological approach to ventilate ARDS patients J Appl Physiol 2008 104 1240 10.1152/japplphysiol.00199.2008 18385298

[B13] Caironi P Cressoni M Chiumello D Ranieri M Quintel M Russo SG Cornejo R Bugedo G Carlesso E Russo R Caspani L Gattinoni L Lung opening and closing during ventilation of acute respiratory distress syndrome Am J Respir Crit Care Med 2010 181 578 586 10.1164/rccm.200905-0787OC 19910610

[B14] Guervilly C Forel J-M Hraiech S Demory D Allardet-Servent J Adda M Barreau-Baumstark K Castanier M Papazian L Roch A Right ventricular function during high-frequency oscillatory ventilation in adults with acute respiratory distress syndrome Crit Care Med 2012 40 1539 1545 10.1097/CCM.0b013e3182451b4a 22511135

[B15] Miranda DR Klompe L Cademartiri F Haitsma JJ Palumbo A Takkenberg JJ Lachmann B Bogers AJ Gommers D The effect of open lung ventilation on right ventricular and left ventricular function in lung-lavaged pigs Crit Care 2006 10 R86 10.1186/cc4944 16764730 PMC1550948

